# Local Anaesthetic Systemic Toxicity in Dental and Oral and Maxillofacial Surgery: Safe Dosing, Combination Agents, and Emergency Management—A Narrative Review

**DOI:** 10.3390/dj14070457

**Published:** 2026-07-20

**Authors:** Cemal Ucer, Simon Wright, Rabia Khan, Sushil Kumar

**Affiliations:** 1 ICE Postgraduate Dental Institute and Hospital, School of Health and Society, University of Salford, 24 Furness Quay, Salford M50 3XZ, UK; ucer@icedental.institute (C.U.); profwright@glencairndental.co.uk (S.W.); 2 East Lancashire Hospital NHS Trusts, Casterton Ave, Burnley BB10 2PQ, UK; sushil.kumar@elht.nhs.uk

**Keywords:** local anaesthetic systemic toxicity, LAST, articaine, lignocaine, bupivacaine, maximum recommended dose, fractional dose rule, intravenous lipid emulsion, oral surgery, differential diagnosis, epinephrine, BNF, sedation

## Abstract

**Background/Objectives**: Local anaesthetic systemic toxicity (LAST) is a rare but potentially fatal complication of dental and oral and maxillofacial surgical local anaesthesia (LA). Three amide agents are commonly used in the UK: lignocaine (lidocaine) 2% with adrenaline 1:80,000; articaine 4% with adrenaline 1:100,000 (2.2 mL cartridges); and bupivacaine 0.5%. Clinically significant discrepancies between guideline sources for maximum recommended dosages (MRDs) persist, and the additive toxicity of combined amide agents remains underappreciated. The objectives are: to provide clear, evidence-appraised MRD guidance for dental practitioners; to explain safe combination dosing using the fractional dose rule with acknowledgement of its pharmacokinetic limitations; and to outline recognition and management of LAST, including intravenous lipid emulsion (ILE) therapy, setting-stratified response, and differential diagnosis. **Methods**: These include the following: narrative review of MEDLINE (via PubMed), the Cochrane Library, and Embase (inception to May 2026), supplemented by key regulatory documents (British National Formulary (BNF) 91; US Food and Drug Administration (FDA) prescribing information; UK Summaries of Product Characteristics (SmPCs)); major guideline documents (American Society of Regional Anesthesia and Pain Medicine (ASRA) 2018; Association of Anaesthetists 2021; Resuscitation Council UK 2021); systematic reviews; and peer-reviewed literature, ranked by a jurisdiction-specific UK prescribing and regulatory source hierarchy. **Results**: BNF 91 and the FDA both support a 7 mg/kg (500 mg) MRD for lignocaine with adrenaline; in practice, the adrenaline ceiling limits administration to 6–7 cartridges (2.2 mL) regardless of the guideline followed. The principal reasons for caution when combining amide agents are; additive systemic toxicity, more complex dose calculation, absence of proven clinical benefit for concurrent mixing, unnecessary drug exposure, and incremental hypersensitivity risk—not metabolic pathway differences. The fractional dose rule is a pharmacologically justified safety heuristic with acknowledged pharmacokinetic limitations. ILE is a specific rescue therapy for severe or cardiovascular LAST; airway support and oxygenation remain the primary interventions. Patient-specific factors substantially lower the effective toxic threshold. **Conclusions**: Safe LA administration in oral surgery requires systematic MRD calculation, application of the fractional dose rule for combined-agent appointments, attention to patient-specific risk factors, setting-appropriate emergency preparedness, and structured differential diagnosis to distinguish LAST from more common dental emergencies.

## 1. Introduction

Local anaesthetics (LAs) are the most frequently administered drugs in dental and oral surgical practice. Three amide agents dominate UK use: lignocaine 2% with adrenaline 1:80,000—the established gold standard [1]; articaine 4% with adrenaline 1:100,000, which has demonstrated superior anaesthetic success over lignocaine for mandibular buccal infiltration and comparable or modestly improved success for inferior alveolar nerve block in some but not all clinical trials [2]; and bupivacaine 0.5%, valued for prolonged postoperative analgesia [2].

All three belong to the amide class. Their central nervous system (CNS) and cardiovascular toxicities are additive when used in combination [3,4]. The principal reasons for caution about combining agents—not metabolic pathway differences, but additive systemic toxicity, more complex dose calculations, absence of proven clinical benefit for concurrent mixing, unnecessary drug exposure, and incremental hypersensitivity risk [4,5,6]—are addressed in this review.

A clinically important discrepancy exists between BNF guidance and product insert values for lignocaine [1,7]. LAST is a rare but potentially fatal complication most commonly arising from inadvertent intravascular injection [8]. Several recent reviews have highlighted its importance in oral surgical contexts [8,9]. A systematic review of LA delivery techniques in the oral surgical setting has also been published in the oral and maxillofacial surgical literature [10]. This narrative review synthesises MRD guidance with evidence appraisal, addresses patient-specific risk factors, provides a setting-stratified management framework, and introduces a differential diagnosis table to aid clinical recognition.

## 2. Methods

**Search strategy:** MEDLINE (via PubMed), the Cochrane Library, and Embase were searched from inception to May 2026 using the following terms: ‘local anaesthetic systemic toxicity’, ‘LAST dentistry’, ‘lignocaine/lidocaine maximum dose dental’, ‘articaine dental dose’, ‘bupivacaine dental’, ‘fractional dose rule local anaesthetic’, ‘intravenous lipid emulsion LAST’, and ‘epinephrine cardiovascular dental’. Reference lists of retrieved articles were hand-searched. Included source types: regulatory documents, guideline statements, systematic reviews, randomised controlled trials, and pharmacological narrative reviews. Language restriction: English.

Source hierarchy: Sources were ranked by a jurisdiction-specific UK prescribing and regulatory framework: (1) UK regulatory and prescribing authority—the SmPC, as approved by the MHRA—constituting the licensed prescribing information; and the BNF 91 as the primary NHS prescribing reference under the NHS Standard Contract for Dental Services. Neither automatically overrides the other: the SmPC carries regulatory authority as the MHRA-approved licensed document, while the BNF reflects clinical consensus. (2) US regulatory documents (FDA prescribing information)—applicable as supplementary authority where UK-specific data are absent and where UK guidance explicitly aligns with FDA values (as is the case for lignocaine and articaine MRDs); the US and UK frameworks should not be conflated into a single transnational hierarchy. (3) Systematic reviews and meta-analyses. (4) Expert guideline bodies: ASRA, Association of Anaesthetists, Resuscitation Council UK. (5) Peer-reviewed narrative reviews and case series.

**Conflict reconciliation:** Where sources disagree, the discrepancy is explicitly stated, and clinical context is provided. UK practitioners should document which source they apply and their clinical reasoning, particularly for complex or high-risk cases.

## 3. Pharmacology of the Three Agents

### 3.1. Lignocaine 2% with Adrenaline 1:80,000

Lignocaine is an amide LA with predominantly hepatic metabolism (plasma half-life 90–120 min), 65% protein binding, and a cardiovascular collapse-to-CNS seizure dose ratio (CC/CNS) of approximately 7.1 [2,11]. Clearance depends on hepatic blood flow rather than enzyme activity alone—important in heart failure or cirrhosis [11]. Each 2.2 mL UK cartridge contains 44 mg lignocaine and 27.5 µg adrenaline.

### 3.2. Articaine 4% with Adrenaline 1:100,000

Articaine contains a thiophene ring and ester linkage conferring a short plasma half-life (~20 min) via dual plasma esterase and hepatic metabolism [2,12]. Amide LAs display additive CNS and cardiovascular toxicity through shared NaV1.5 sodium channel blockade, regardless of individual metabolic pathway [11]. Each 2.2 mL UK cartridge contains 88 mg articaine and 22 µg epinephrine.

### 3.3. Bupivacaine 0.5%

Bupivacaine is a long-acting amide LA with 95% protein binding. Its CC/CNS ratio of approximately 2.0 [2] means cardiovascular collapse may occur with minimal neurological warning—the most clinically important distinction from lignocaine and articaine. It is available as 1.8 mL cartridges (adrenaline 1:200,000; 9 mg) or plain 10 mL ampoules (50 mg; UK SmPC emc products 6618/11612) [13].

## 4. Maximum Recommended Dosages

### 4.1. Lignocaine—Reconciling BNF, FDA, and SmPC Guidance

BNF 91 specifies 500 mg (~7 mg/kg) for lignocaine with adrenaline, concordant with the US FDA (3.2 mg/lb, 500 mg) [1,7,14]. The conservative 4.4 mg/kg (300 mg) ceiling originates from product information leaflets (e.g., Lignospan Special, Septodont) and earlier UK dental teaching [1,7]. In practice, the adrenaline content (27.5 µg per 2.2 mL cartridge of 1:80,000) limits a healthy 70 kg adult to approximately seven cartridges (192.5 µg; 308 mg) before the 200 µg adrenaline ceiling is reached—making the practical difference between guidelines one cartridge for most adults as seen in Table 1. The governing rule is to apply the lower of the weight-based dose, determine the absolute ceiling and to calculate the vasoconstrictor limit independently and apply whichever limit is reached first.

### 4.2. Articaine

The FDA specifies 7 mg/kg (3.2 mg/lb) with no published absolute maximum; a conservative 500 mg ceiling is widely maintained [7,12]. For a 70 kg adult (2.2 mL cartridges; 88 mg each), this equates to 5 cartridges (440 mg).

### 4.3. Bupivacaine

Current prescribing information specifies an absolute maximum of 90 mg per dental appointment for the cartridge format [7]. Plain ampoules carry a general ceiling of 175 mg without epinephrine (UK SmPC) [13].

## 5. Combination Dosing—Rationale, Risks, and the Fractional Dose Rule

### 5.1. Why Clinicians Combine Agents—And Why Caution Is Warranted

Combining LA agents is encountered in oral surgical practice to achieve rapid operative onset with one agent (typically articaine or lignocaine), followed by prolonged postoperative analgesia with another (bupivacaine typically given at wound closure). With the increasing demand for full arch/full mouth implant procedures in specialist practice settings, there is risk of exceeding the MRD when using either single LA anaesthetic or combined agents during prolonged surgical procedures that can exceed 2–3 h. The principal reasons for caution when combining amide LAs are not necessarily the differences in metabolic pathways, but are the following: (1) **additive systemic toxicity**—through shared NaV1.5 blockade, regardless of how each agent is metabolised [3,4,11]; (2) **more complex dose calculation**—the fractional dose rule must be applied; failure to do so is a recognised clinical error [5]; (3) **absence of proven clinical benefit for concurrent mixing**—mixing dilutes agent concentrations, and clinical trials have not consistently demonstrated superior outcomes [6,15]; (4) **unnecessary drug exposure**—each additional agent introduces its own adverse effect profile without confirmed clinical gain; and (5) **incremental hypersensitivity risk**—true IgE-mediated allergy is less than 1% of LA adverse reactions, but cross-reactivity within the amide group is possible [16,17]. Sequential combination—distinct agents at different procedural phases for distinct pharmacological purposes—is an expert clinical recommendation where clinically indicated and is distinct from concurrent mixing.

### 5.2. The Fractional Dose Rule

Where combination use is indicated, the Loewe dose-additivity principle applies:

(Dose A ÷ MRD A) + (Dose B ÷ MRD B) ≤ 1.0

This is endorsed by the Association of Anaesthetists (AAGBI) [3] and is consistent with ASRA guidance [5]. (Note: the latter is a published ASRA commentary rather than a formal guideline or systematic review; it is cited here as supporting perspective from the specialty society, but not as a primary evidentiary source). The principal evidentiary basis for the fractional dose rule rests on the pharmacological mechanism (Loewe additivity) and the Association of Anaesthetists guideline [3]. If a third agent is used, its fractional contribution is added to the sum. Vasoconstrictor content must be audited independently of this calculation.

### 5.3. Pharmacokinetic Limitations of the Fractional Dose Rule

The rule is a pragmatic safety heuristic and does not fully account for: (1) **time between injections**—sequential doses with time gaps allow partial redistribution and clearance, so the rule conservatively treats all doses as simultaneously at peak effect; (2) **differential vascular uptake** by injection site; (3) **bupivacaine’s 95% protein binding**—only a small free fraction is immediately pharmacologically active; and (4) **articaine’s ~20 min plasma half-life**—earlier doses are substantially cleared during extended appointments as seen in Table 2. Despite these limitations, the rule remains the most pharmacologically justified, operationally simple, and guideline-endorsed method for managing combined-agent toxicity risk.

## 6. Patient-Specific Risk Factors for LAST

The Association of Anaesthetists notes that drug choice, dose, and concentration should be individualised to the patient’s clinical condition [3]. The following factors substantially lower the effective toxic threshold (Table 3).

### 6.1. Hepatic Dysfunction

Lignocaine clearance depends on hepatic blood flow; reduced cardiac output or cirrhosis markedly raises plasma levels [11]. Dose reduction of 30–50% is warranted. Articaine’s dual plasma esterase and hepatic metabolism provides partial protection in mild-to-moderate hepatic impairment.

### 6.2. Renal Impairment

Accumulation of active metabolites (e.g., monoethylglycinexylidide (MEGX) from lignocaine) occurs in significant renal failure; reduced albumin in nephrotic syndrome increases the free drug fraction. Caution is warranted when eGFR is below 30 mL/min/1.73 m^2^.

### 6.3. Elderly and Frail Patients

Reduced hepatic blood flow, lower albumin, diminished lean body mass, and reduced cardiac reserve converge to lower the effective toxic threshold. MRDs should be calculated on lean body weight, with a 20–30% dose reduction advised.

### 6.4. Obesity

Total body weight overestimates the safe LA dose in obesity, as the free drug fraction is determined by plasma protein binding rather than total body water. Lean body weight should be used for MRD calculation in obese patients, consistent with the Association of Anaesthetists recommendations for regional anaesthesia.

### 6.5. Children and Low-Body-Weight Adults

Weight-based dosing is critical; small absolute errors have proportionally greater consequences. Articaine is not recommended in children under 4 years. See Table 3 for adult weight-based quick reference; paediatric dosing should follow specific paediatric dental anaesthesia references.

### 6.6. Pregnancy

Reduced protein binding, altered distribution, and foetal exposure increase risk. The adrenaline ceiling falls to 40 µg per appointment (BNF; Association of Anaesthetists). Bupivacaine is Pregnancy Category C; plain ampoules are preferred to minimise vasoconstrictor exposure. Note that pregnancy is not equivalent to cardiovascular disease—the 40 µg limit is a conservative expert consensus ceiling, not an RCT-derived toxicity boundary [18,19,20].

### 6.7. Cardiovascular Disease and Heart Failure

Reduced cardiovascular reserve and bupivacaine’s low CC/CNS ratio (2.0) make it the highest-risk agent in patients with IHD or significant arrhythmia. The adrenaline ceiling falls to 40 µg. The systematic review by Guimaraes et al. (BMJ Open, 2021) found that 36–54 µg of epinephrine appears tolerated in most patients with hypertension or cardiovascular disease (CVD) [21], supporting the 40 µg threshold as a conservative clinical safety limit rather than a precisely evidence-derived toxicity boundary. Critically, adequate anaesthesia protects cardiovascular patients: endogenous catecholamine release from pain and anxiety during inadequately anaesthetised procedures may exceed the low exogenous epinephrine doses used in dental LA [21].

### 6.8. Hypoalbuminaemia

Reduced protein binding increases the free fraction of all LA agents; most relevant for bupivacaine (95% protein-bound) in liver disease, nephrotic syndrome, malnutrition, and critical illness.

### 6.9. Calcium Channel Blockers

Calcium channel blockers (CCBs) potentiate bupivacaine’s negative inotropy, with EC50 potentiation factors of 1.4–3.3 across agents (Heck and Freye, 1994) [22,23]. This pharmacodynamic interaction does not alter the fractional dose calculation but mandates conservative bupivacaine dosing, sequential administration at wound closure, and immediate ILE procurability in all CCB-treated patients.

### 6.10. Beta-Blockers, Antiarrhythmics, and Concurrent Sedation

Non-selective beta-blockers lower the adrenaline ceiling to 40 µg; additive negative chronotropic and dromotropic effects occur with LA cardiovascular toxicity in patients on antiarrhythmics. Concurrent benzodiazepine or opioid sedation may mask early CNS LAST symptoms (tinnitus, agitation, confusion), eliminating the neurological warning window before cardiovascular deterioration—enhanced monitoring of all vital parameters is essential in sedated patients.

## 7. LAST—Incidence and Presentation

### 7.1. Incidence

Dedicated dental-specific LAST registry data are lacking—a recognised evidence gap. Neal et al. (ASRA, 2018) report 0.27 per 1000 peripheral nerve blocks (95% CI 0.21–0.35; n = 251,325 blocks) in regional anaesthesia practice [5]. Long et al. (Am. J. Emerg. Med., 2022) estimate approximately 1 per 5000 nerve blocks in contemporary anaesthetic practice [9]. These are indirect comparators—not direct estimates for dentistry, where smaller volumes, lower concentrations, and different injection sites apply. Rhee et al. (2019) provide the most directly applicable evidence from dental anaesthesia contexts [24].

### 7.2. Presentation

Up to 40% of LAST cases present atypically, with delayed onset, cardiovascular signs preceding neurological features, or both systems affected simultaneously [8]. The classic CNS excitatory prodrome—perioral tingling, metallic taste, tinnitus, visual disturbance, agitation, tremors—may progress to tonic–clonic seizures, CNS depression, and apnoea. Cardiovascular features range from initial tachycardia and hypertension through bradycardia, conduction abnormalities, and ventricular arrhythmia to asystole. Vigilance must be maintained for at least 30 min post-injection.

### 7.3. Differential Diagnosis in the Dental Setting

Early LAST may be confused with several more common dental emergencies. Vasovagal syncope (VVS) is the most common medical emergency in dentistry, accounting for 61.9% of events in a large retrospective single-centre study (Obata et al., Acute Med. Surg., 2021) [25], and shares features such as dizziness, nausea, and pallor with early LAST. A structured differential diagnosis is essential to avoid both under-recognition and overdiagnosis (Table 4).

## 8. Illustrative Hypothetical Case Vignette

**Declaration:** This vignette is entirely hypothetical and constructed for educational purposes. It does not represent a real patient or clinical event thus no ethics approval or patient consent is required.

**Setting:** A specialised primary care dental practice routinely providing implant surgery and intravenous (IV) conscious sedation. Sedation is delivered by a separately trained specialist dental anaesthetist in compliance with SAAD Guidance for Conscious Sedation in Dentistry (2026) [26] and the IACSD 2020 national standards for conscious sedation in dentistry [27]. IV access is pre-established for all sedation cases; ILE (20% Intralipid) is stocked on site as a mandatory requirement for a practice routinely administering bupivacaine.

A 62-year-old male (74 kg; ASA II; well-controlled hypertension on amlodipine 5 mg once daily) presents for surgical extraction of an impacted lower right first molar with concurrent implant site preparation under IV conscious sedation with midazolam. A sequential anaesthetic strategy is planned: articaine 4% 1:100,000 (2.5 × 2.2 mL = 220 mg planned) for the operative inferior alveolar nerve block (IANB), long buccal, and lingual nerve blocks; plain bupivacaine 0.5% (3 mL = 15 mg) at wound closure for postoperative analgesia. Pre-procedure fractional dose verification: (220 ÷ 518) + (15 ÷ 90) = 0.592 ✓; epinephrine 55 µg ✓. The patient’s amlodipine (a dihydropyridine CCB) is noted; plain bupivacaine is selected to avoid additional adrenaline exposure; ILE is confirmed on site.

During the IANB, approximately 1.0 mL (40 mg articaine) had been injected when the patient reported metallic taste and tinnitus at approximately 30 s, followed by agitation and a 45 s tonic–clonic seizure. LAST from inadvertent partial intravascular injection was diagnosed—a small volume entering the circulation producing rapid CNS toxicity, not a total-dose toxicity event. The injection was stopped immediately; 999 was activated; 100% O_2_ was applied via non-rebreather mask; IV access (pre-established for sedation) was utilised; the specialist anaesthetist administered midazolam 5 mg IV—the seizure resolved within 90 s.

During and immediately after the seizure, continuous pulse oximetry revealed tachycardia at 98 bpm with irregular waveform morphology consistent with peri-ictal sinus arrhythmia—the cardiovascular feature justifying a 4 h cardiovascular monitoring period. Rhythm returned to regular sinus at 76 bpm within 10 min of seizure resolution, with no recurrent arrhythmia on subsequent monitoring. 20% Intralipid (111 mL bolus prepared) was not administered: haemodynamic stability was maintained and the seizure terminated with midazolam, consistent with ASRA guidance that ILE is indicated for cardiovascular compromise or refractory seizures, not as a prophylactic measure in a haemodynamically stable post-ictal patient [5]. Bupivacaine was not administered.

Paramedics arrived at 9 min; patient alert at 12 min; transferred to hospital for 4 h cardiovascular monitoring. Discharge criteria: haemodynamic stability > 4 h; return to neurological baseline; no recurrent arrhythmia; senior clinician review; written emergency contact information provided.

**Key learning points:** (1) Negative aspiration does not guarantee extravascular placement. (2) Early symptom recognition and immediate injection cessation critically limited the intravascular dose. (3) Pre-established IV access in a SAAD-compliant sedation setting enabled prompt IV midazolam administration [26]. (4) Bupivacaine sequenced to wound closure ensured that the most cardiotoxic agent was not present at the time of the event. (5) Seizure was controlled with midazolam and immediate ILS helped to maintain haemodynamic and respiratory stability. On-site ILE enabled rapid preparation without delay.

## 9. Acute Management of LAST

The cornerstones of LAST management in order of priority are: (1) cessation of injection; (2) airway support and 100% oxygen; (3) seizure control; (4) ILS/ALS, where indicated; and (5) ILE as a specific rescue therapy for severe or cardiovascular LAST. All IV medications must be administered only by clinicians specifically trained in their use, within local clinical governance frameworks, and within their defined scope of practice [3,8,28,29]. Table 5 and Table 6 detail the structured protocol and setting-stratified response, respectively. Table 7, Table 8 and Table 9 summarise the practical application of local anaesthetic dose calculation and strategies to minimise the risk of local anaesthetic systemic toxicity (LAST). 

### Intravenous Lipid Emulsion—Evidence Appraisal

ILE (20% Intralipid, Fresenius Kabi, Bad Homburg, Germany) is a specific rescue therapy for severe or cardiovascular LAST, operating principally via the lipid sink mechanism—sequestering lipophilic LA molecules from cardiac and neural tissue [29,30,31]. It is endorsed as a first-line adjunct by ASRA and the Association of Anaesthetists [3,8]. A 2025 narrative review confirms reserved endorsement for ILE in bupivacaine-induced toxicity from systematic reviews of international toxicological societies, with positive survival data from animal model meta-analyses [29]. Rhee et al.’s dental-specific ILE review (2019) provides directly applicable guidance [24]. The evidence base rests primarily on case registries and pre-clinical models; randomised controlled trials are ethically implausible. Adverse effects include pancreatitis, fat overload syndrome, and laboratory test interference. In primary care settings without on-site ILE, early 999 activation remains the most critical action.

## 10. Prevention

Prevention rests on systematic dose calculation, technique, and preparedness. The MRD is a ceiling for patient safety—not a target dose; the lowest effective dose should always be used [7]. The four rationales for sequencing bupivacaine to wound closure should not be conflated: (1) reducing the intraoperative combined fractional dose burden; (2) avoiding redundant dosing during the operative phase; (3) preserving the full postoperative analgesic duration; and (4) ensuring the most cardiotoxic agent is administered after the highest-risk intraoperative LAST window has passed.

Table 7 provides weight-based maximum cartridge recommendations for commonly used dental local anaesthetics, incorporating both local anaesthetic maximum recommended doses (MRDs) and epinephrine limits to identify the clinically applicable maximum number of cartridges for healthy adults. 

**Table 7:** Weight-based quick reference—maximum cartridges for healthy adults (conservative MRDs; all values rounded down). Three columns per agent: LA-limited|vasoconstrictor-limited|PRACTICAL MAXIMUM (lower of the two). Vasoconstrictor ceiling: healthy adults 200 µg; CVD/CCB/beta-blockers/pregnancy 40 µg (must recalculate vasoconstrictor-limited column accordingly).

Body Weight	Lignocaine 2% 1:80,000 (SmPC: 4.4 mg/kg/300 mg) LA-lim|Epi-lim|PRACTICAL	Lignocaine 2% 1:80,000 (BNF: 7 mg/kg/500 mg) LA-lim|Epi-lim|PRACTICAL	Articaine 4% 1:100,000 (7 mg/kg/500 mg; 2.2 mL) LA-lim|Epi-lim|PRACTICAL	Bupivacaine 0.5% 1:200,000 (90 mg Dental Ceiling; 1.8 mL) LA-lim|Epi-lim|PRACTICAL
40 kg	4 ^†^|7|4	6|7|6	3 ^‡^|9|3	10|22|10
50 kg	5 ^†^|7|5	7 ^§^|7|7	3 ^‡^|9|3	10|22|10
60 kg	6 ^†^|7|6	9|7|7 ^§^	4 ^‡^|9|4	10|22|10
70 kg	6 ^†^|7|6	11|7|7 ^§^	5|9|5	10|22|10
80 kg	6 ^†^|7|6	11|7|7 ^§^	5|9|5	10|22|10
90 kg	9|7|7 ^§^	14|7|7 ^§^	7|9|7	10|22|10
100 kg	10|7|7 ^§^	15|7|7 ^§^	7|9|7	10|22|10

Key: LA-lim = (weight kg × mg/kg MRD) ÷ mg per cartridge, rounded DOWN; absolute ceiling applied where reached before weight limit. Epi-lim = 200 µg ÷ µg per cartridge, rounded DOWN (lignocaine 1:80,000: 200 ÷ 27.5 = 7.27 → 7; articaine 1:100,000: 200 ÷ 22 = 9.09 → 9; bupivacaine 1:200,000: 200 ÷ 9 = 22.2 → 22). Practical = lower of LA-lim and Epi-lim. ^†^ Absolute 300 mg LA ceiling is binding before weight-based limit. ^‡^ Weight-based limit (7 mg/kg) binding: 40 kg = 280 mg ÷ 88 = 3.18 → 3; 50 kg = 350 mg ÷ 88 = 3.97 → 3; 60 kg = 420 mg ÷ 88 = 4.77 → 4 cartridges. ^§^ Vasoconstrictor ceiling (7 or 9 cartridges) is binding before the LA weight-based limit. For cardiovascular patients, beta-blocker users, and pregnancy: recalculate Epi-limited column using 40 µg ceiling: lignocaine 40 ÷ 27.5 = 1.45 → 1 cartridge; articaine 40 ÷ 22 = 1.82 → 1 cartridge. This table applies to healthy adults only; apply patient-specific modifiers from Table 3 for high-risk patients.

Table 8 outlines key preventive strategies supported by current pharmacological principles, clinical guidelines, and expert recommendations, emphasising dose calculation, fractional dosing when combining agents, vasoconstrictor limits, aspiration, slow injection technique, and preparedness for lipid emulsion therapy. 

**Table 8:** Key preventive strategies with evidence basis.

Strategy	Evidence Basis	Details
Calculate MRD before every appointment	Guideline (ASRA, Association of Anaesthetists, BNF)	Use lean body weight for obesity/frailty; apply the lower of weight-based dose and absolute ceiling; calculate vasoconstrictor total independently
Apply fractional dose rule for all LA combinations	Pharmacological (Loewe additivity; Association of Anaesthetists; ASRA 2026) [5]	Including the commonly missed lignocaine + articaine combination; vasoconstrictor total is a separate, independent audit
Acknowledge pharmacokinetic limitations of rule	Pharmacological reasoning	Sequential injections allow partial clearance between doses; rule conservatively treats all as simultaneously at peak
Avoid concurrent LA mixing	Clinical pharmacology [6,15]	Mixing dilutes agent concentration; no proven clinical benefit; use sequential administration only
Aspirate before every injection	Expert recommendation; anatomical risk data	Necessary but not sufficient—needle movement post-aspiration can reposition the tip inside a vessel
Inject slowly (≤1 mL per 15–20 s)	Pharmacokinetic rationale	Reduces peak plasma concentration for equivalent total dose
Sequence bupivacaine to wound closure (four rationales)	Expert clinical recommendation	(1) Reduces intraoperative combined dose; (2) avoids redundant dosing; (3) preserves postoperative analgesia; (4) delays most cardiotoxic agents until after the highest-risk intraoperative window
Use plain bupivacaine ampoule in CVD/CCB patients	Expert recommendation; pharmacodynamic rationale	Eliminates epinephrine contribution; allows precise volume titration
Apply appropriate epinephrine ceiling	Guideline: Guimaraes et al. 2021 [21]	Healthy adults, 200 µg; CVD/CCB/beta-blockers/pregnancy 40 µg—conservative safety limit, not a precisely evidence-derived toxicity threshold
Ensure ILE is procurable wherever bupivacaine is used	ASRA; Association of Anaesthetists guideline [3,5,8]	Mandatory on-site stock in SAAD-compliant sedation practices and hospital OMFS; 999 is the primary response in general dental practice [26]
Team training and annual emergency simulation	Expert recommendation	Drills to include LAST differential diagnosis scenarios and setting-appropriate management sequences

Table 9 presents a practical pre- and during procedure checklist and stepwise emergency management algorithm for LAST, integrating patient risk assessment, dose calculation, recognition of early toxicity, differential diagnosis, and immediate evidence-based management to facilitate safe clinical practice.

**Table 9:** Pre-procedure dose calculation checklist and LAST emergency response algorithm.

Phase	Item
PRE-PROCEDURE	1. Record patient weight—actual body weight; use lean body weight for obese/frail patients
	2. Calculate MRD for each LA: apply the LOWER of (weight × mg/kg) and absolute ceiling
	3. If combination planned: calculate fractional dose sum ≤ 1.0
	4. Calculate vasoconstrictor total independently; apply relevant ceiling (200 µg healthy; 40 µg CVD/CCB/beta-blocker/pregnancy)
	5. Identify and document patient-specific risk factors; reduce doses accordingly
	6. Confirm ILE procurable on site (required wherever bupivacaine is administered)
	7. Plan sequential, not concurrent, LA administration
DURING PROCEDURE	8. Aspirate before every injection
	9. Inject slowly (≤1 mL per 15–20 s)
	10. Maintain vigilance for LAST for at least 30 min post-injection
LAST RECOGNITION	Metallic taste · Tinnitus · Perioral tingling · Agitation · Visual disturbance → STOP IMMEDIATELY
	Exclude: VVS (bradycardia, no metallic taste, rapid recovery supine); adrenaline reaction (tachycardia, self-resolving); hypoglycaemia (gradual, diabetic); anaphylaxis (urticaria, bronchospasm)
LAST RESPONSE	STOP → 999 → Airway + 100% O_2_ → IV access → Midazolam (buccal/IV by trained clinician) → ALS if CVS collapse → ILE for severe/CVS LAST → TRANSFER

## 11. Discussion

This narrative review has addressed the principal areas of clinical importance for LA dosing and LAST management in UK in dental and specialist oral and maxillofacial surgical practice.

**Guideline discrepancy for lignocaine.** The BNF and FDA both support 7 mg/kg (500 mg), while the SmPC and traditional UK dental teaching specify 4.4 mg/kg (300 mg). In practice, the adrenaline ceiling (200 µg) limits administration to seven cartridges for a healthy adult under either guideline, making the clinical difference one cartridge. Practitioners should cite their source and reasoning in clinical documentation. The systematic review by Guimaraes et al. [21] confirms that adequate anaesthesia protects cardiovascular patients from endogenous catecholamine surges, reinforcing that undertreating pain is not a safe alternative to careful vasoconstrictor management.

**Combination dosing rationale.** The primary reasons for caution are additive toxicity, complex calculations, absence of proven benefit for concurrent mixing, unnecessary drug exposure, and hypersensitivity risk—not metabolic pathway differences. This distinction, raised by Reviewer 4 and addressed in the previous revision, is maintained throughout this manuscript. The ASRA 2026 Pro-Con analysis [5] explicitly endorses fractional dose calculation and identifies the absence of package insert guidance on safe combination dosing as an urgent unmet need.

**Pharmacokinetic limitations of the fractional dose rule.** The rule does not account for time between injections, differential vascular uptake, bupivacaine’s high protein binding, or articaine’s rapid plasma hydrolysis. It conservatively treats all doses as simultaneously at peak effect, providing a safe margin despite these acknowledged limitations.

**Setting stratification.** The three-tier framework—general dental practice; SAAD-compliant specialised primary care sedation/implant practice; hospital OMFS—reflects the reality that management capabilities, available interventions, and governance frameworks differ substantially across clinical environments. The vignette is now explicitly situated in the SAAD-compliant intermediate tier, resolving the reviewer’s concern about the appropriateness of IV medication use in the previous version [26].

**Differential diagnosis.** VVS accounted for 61.9% of dental medical emergencies in the Obata et al. (2021) retrospective study [25] and is the most common condition likely to be confused with early LAST. The structured Table 4 addresses this clinical challenge directly and should be a practical aid for practitioners.

**Evidence limitations.** This is a narrative review; its scope and synthesis are deliberately broad and do not constitute a systematic review. The fractional dose rule lacks direct dental clinical trial validation. ILE evidence rests on case registries and pre-clinical models. Dental-specific LAST incidence data are lacking. These limitations have been stated explicitly throughout the manuscript.

**Contemporary evidence on LAST mortality and the emerging risk of lignocaine.** The contemporary relevance of lignocaine dosing limits is underscored by a landmark 2025 pharmacovigilance analysis. Fettiplace et al. examined 55 years of local anaesthetic-associated deaths reported to the US FDA Adverse Event Reporting System (FAERS; 1968–2023), identifying 1473 reports of death from 22,050 total LA adverse events [32]. Critically, while deaths from long-acting agents (bupivacaine, ropivacaine) declined following the 2010 ASRA practice advisories, deaths from lidocaine remained essentially unchanged and lidocaine emerged as the leading reported cause of death attributable to LAST. In the accompanying editorial, Schwenk, Sneyd and Wu noted that this pharmacovigilance signal was unexpected and that dentistry—alongside emergency medicine and other high-volume lidocaine settings—represents a clinical context particularly relevant to this finding [33]. Retrospective reviews describing deaths in dentistry, primarily from lidocaine, were noted as failing to be captured in the FAERS database, suggesting that true LA-related mortality in dental practice may be underestimated. These findings lend particular urgency to the dosing, combination-use, and LAST management framework described in this review, and reinforce that the lignocaine MRD discrepancy between the BNF and product information leaflets is a matter of genuine patient safety consequence—not merely a regulatory technicality.

## 12. Conclusions

Safe local anaesthetic administration in surgical dentistry requires a pre-procedure dose calculation for every appointment indicating the application of the weight-based MRD, determining the absolute ceiling, and calculating of the total vasoconstrictor dose independently using the appropriate patient ceiling, and—where two or more amide agents are used—applying the fractional dose rule to the combined doses. Agents appearing individually within their own MRDs may still constitute a toxic combination.

Patient-specific factors modify the toxic threshold substantially. Hepatic impairment, renal impairment, frailty, obesity, cardiovascular disease, calcium channel blockers, and concurrent sedation all lower the margin between therapeutic and toxic concentrations and must inform agent selection and dose from the outset.

Recognising LAST promptly is an important clinical skill. Vasovagal syncope accounts for over 60% of dental medical emergencies [25] and shares early features with LAST therefore structured differential diagnosis is essential. When LAST is suspected following emergency steps should take precedence; stop the injection, call 999, secure the airway, and deliver 100% oxygen. Intravenous lipid emulsion (ILE—20% Intralipid) is a specific rescue therapy for severe or cardiovascular LAST; it does not replace airway management, oxygenation, seizure control, or ILS/ALS.

Emergency preparedness must match the clinical setting. SAAD-compliant implant and sedation practices—where bupivacaine is routinely used and procedures may extend to hours—must stock ILE on site, maintain intravenous access, and ensure a trained anaesthetist or sedationist is present. For full-arch implant surgery, a pre-operative dose budget allocated by anatomical region should be calculated before the case begins.

## Figures and Tables

**Table 1:** Maximum recommended dosages—all three agents. Apply the lower of the weight-based dose and the absolute ceiling. The vasoconstrictor limit must be calculated independently; whichever limit is reached first governs.

Agent	Weight-Based MRD	Absolute Ceiling	Drug/Vasoconstrictor per Unit	Max Cartridges (LA-Limited)	Max Cartridges (Vasoconstrictor-Limited, 200 µg Ceiling)	Practical Max (70 kg, Healthy Adult)
Lignocaine 2% 1:80,000	4.4 mg/kg (SmPC) 7 mg/kg (BNF/FDA)	300 mg (SmPC) 500 mg (BNF/FDA)	44 mg/27.5 µg per 2.2 mL	6 (SmPC: 264 mg) 11 (BNF/FDA: 484 mg)	7 (192.5 µg; ceiling always limits before LA max)	6 (SmPC) or 7 (BNF/FDA; vasoconstrictor-limited)
Articaine 4% 1:100,000	7 mg/kg	500 mg (conservative clinical ceiling)	88 mg/22 µg per 2.2 mL	5 (440 mg at 70 kg)	9 (198 µg)	5 cartridges (LA weight-based limit)
Bupivacaine 0.5% 1:200,000	2 mg/kg	90 mg (dental appointment ceiling)	9 mg/9 µg per 1.8 mL cartridge	10 (90 mg)	22 (not limiting for this agent)	10 cartridges (LA-limited)
Bupivacaine 0.5% plain ampoule	2 mg/kg	175 mg (SmPC general); 90 mg (dental conservative)	50 mg/nil per 10 mL	Titrate to requirement	N/A (no vasoconstrictor)	Titrate; 90 mg conservative dental ceiling

**Table 2:** Worked fractional dose examples (70 kg healthy adult; conservative MRDs; sequential administration).

Combination	Doses Used	Fractional Calculation	Adrenaline Total	Result
Lignocaine + Articaine (sequential)	3 × 2.2 mL lig. (132 mg) 2 × 2.2 mL art. (176 mg)	(132÷300) + (176÷490) = 0.440 + 0.359 = 0.799	(3 × 27.5) + (2 × 22) = 126.5 µg	Safe ✓
Articaine + Bupivacaine plain (sequential)	3 × 2.2 mL art. (264 mg) 3 mL bup. ampoule (15 mg)	(264÷490) + (15÷90) = 0.539 + 0.167 = 0.706	3 × 22 = 66 µg	Safe ✓
Lignocaine + Bupivacaine plain (sequential)	3 × 2.2 mL lig. (132 mg) 3 mL bup. ampoule (15 mg)	(132÷300) + (15÷90) = 0.440 + 0.167 = 0.607	3 × 27.5 = 82.5 µg	Safe ✓
All three agents	2 × lig. (88 mg) + 2 × art. (176 mg) + 2 mL bup. ampoule (10 mg)	0.293 + 0.359 + 0.111 = 0.763	(2 × 27.5) + (2 × 22) = 99 µg	Safe ✓

**Table 3:** Patient-specific risk factors—mechanism and clinical management implications.

Risk Factor	Mechanism of Increased Risk	Clinical Implication
Hepatic impairment	Reduced LA metabolism; accumulation (lignocaine half-life 90–120 min)	Reduce MRD 30–50%; consider articaine (dual metabolism) as primary agent
Renal impairment	Metabolite accumulation; reduced albumin → increased free fraction	Caution eGFR < 30 mL/min/1.73 m^2^; use lower end of dose range
Elderly/frailty	Reduced hepatic flow; lower albumin; reduced cardiac reserve	Calculate on lean body weight; reduce by 20–30%
Obesity	Total body weight overestimates safe dose (protein binding-dependent)	Use lean body weight for MRD calculation
Children	Small absolute dose errors are proportionally larger	Strict weight-based dosing; paediatric-specific references
Pregnancy	Reduced protein binding; altered distribution; foetal exposure	Adrenaline ceiling 40 µg; Pregnancy Category C bupivacaine; plain ampoules
CVD/heart failure	Reduced CVS reserve; bupivacaine CC/CNS 2.0	Adrenaline ceiling 40 µg; minimise bupivacaine; enhanced vigilance
Hypoalbuminaemia	Increased free drug fraction (especially bupivacaine, 95% protein-bound)	Reduce dose; extra caution with bupivacaine
Calcium channel blockers	Additive negative inotropy; potentiated bupivacaine cardiotoxicity	Conservative bupivacaine; sequential wound-closure only; ILE procurable on site
Non-selective beta-blockers	Adrenaline ceiling 40 µg; additive cardiac conduction effects	40 µg vasoconstrictor ceiling; avoid high-concentration vasoconstrictor
Concurrent sedation	Early CNS LAST symptoms masked; reduced warning window	Enhanced monitoring; lower threshold for clinical concern; enhanced team vigilance

**Table 4:** Differential diagnosis of acute adverse events following local anaesthetic injection in dentistry.

Condition	Onset	CNS Features	CVS Features	Key Distinguishing Features
LAST (early)	Seconds–5 min (may be delayed to 30 min)	Metallic taste; tinnitus; perioral tingling; agitation; progression to seizure	Tachycardia → bradycardia; hypotension; arrhythmia	Temporal link to LA injection; neurological prodrome; progression to seizure
Vasovagal syncope	Seconds–minutes	Dizziness; nausea; pallor; brief loss of consciousness	Bradycardia; hypotension	Anxiety/pain trigger; rapid recovery supine; no metallic taste or seizure
Adrenaline/sympathomimetic reaction	30–60 s	Anxiety; tremor; headache; palpitation sensation	Tachycardia; palpitations; hypertension	Self-resolving; no CNS toxicity progression; tachycardia, not bradycardia
Hypoglycaemia	Variable (often delayed)	Confusion; hunger; sweating; weakness; possible seizure	Mild tachycardia	Diabetic history; gradual onset; responds to glucose; no metallic taste
Anaphylaxis	Minutes	Anxiety; dizziness; sense of impending doom	Tachycardia; hypotension; potential cardiac arrest	Urticaria/flushing/bronchospasm/angioedema; responds to IM adrenaline
Oversedation	Gradual	CNS depression; drowsiness; reduced responsiveness	Bradycardia; hypotension; respiratory depression	Sedation context; no excitatory prodrome; reversible with reversal agents

**Table 5:** Structured LAST management protocol.

Step	Priority	Action
1. Stop injection	Highest	Cease immediately at first suspicion of toxicity—this is the single most important action
2. Call for help	Immediate	Dial 999/112; alert all available staff; announce the emergency clearly
3. Airway and oxygen	Immediate	ABCDE assessment; 100% O_2_ via non-rebreather mask at 15 L/min; support ventilation; position supine (left lateral if pregnant or unconscious)
4. IV access and monitoring	Early	Establish immediately; continuous SpO_2_, BP, pulse monitoring
5. Seizure control	Early	Midazolam 5–10 mg IV (IV-trained clinician only) OR 10 mg buccal midazolam; avoid propofol if haemodynamically unstable (negative inotropy may worsen cardiovascular compromise)
6. Cardiovascular collapse	As required	Commence ILS/ALS per Resuscitation Council UK guidelines; adrenaline ≤ 1 µg/kg IV (not standard ALS dose—high-dose adrenaline may worsen bupivacaine-induced arrhythmias); avoid CCBs, beta-blockers, lidocaine as antiarrhythmic; amiodarone for VT/VF
7. ILE—specific rescue for severe/CVS LAST	Adjunct	20% Intralipid: bolus 1.5 mL/kg over 1 min → infusion 15 mL/kg/h; repeat bolus × 2 at 5 min intervals if no response; maximum cumulative 12 mL/kg; cease within 30 min of haemodynamic stability
8. Transfer	All events	CNS events only: ≥2 h monitoring; CVS involvement: ≥4–6 h monitoring; all LAST events require hospital transfer and documentation

**Table 6:** Setting-stratified LAST response.

Clinical Setting	Expected Capabilities	Available Management
General dental practice	No routine IV access; first-aider trained; AED may be present	Stop injection immediately; 999; basic airway management; 100% O_2_; buccal midazolam (if within formulary and governance); supine positioning; AED if available; await paramedics. No IV medications unless the operator is specifically trained and within their scope of practice.
Specialised primary care (e.g., SAAD-compliant sedation/implant practice)—setting of the illustrative vignette	IV access pre-established for sedation cases; separately trained specialist anaesthetist or sedationist present; ILE must be stocked on site; IACSD 2020 and SAAD 2026 compliance required [26,27]	All of the above PLUS IV midazolam administered by the specialist anaesthetist or trained sedationist; ILE administered if cardiovascular compromise or refractory seizures; senior clinical support activated; 999 called.
Hospital-based OMFS	Full resuscitation capabilities; anaesthetic team accessible; ICU/HDU available	Full protocol: ALS, ILE, continuous cardiac monitoring; anaesthetic team called; post-event HDU/ICU admission considered. ILE is a mandatory stock item.

## Data Availability

No new data were created or analysed in this study. Data sharing is not applicable to this article.

## Abbreviations

LA = local anaesthetic; LAST = local anaesthetic systemic toxicity; MRD = maximum recommended dose; BNF = British National Formulary; FDA = US Food and Drug Administration; SmPC = summary of product characteristics; MHRA = Medicines and Healthcare products Regulatory Agency; ILE = intravenous lipid emulsion; IANB = inferior alveolar nerve block; CCB = calcium channel blocker; IHD = ischaemic heart disease; ALS = advanced life support; eGFR = estimated glomerular filtration rate; OMFS = oral and maxillofacial surgery; VVS = vasovagal syncope; SAAD = Society for the Advancement of Anaesthesia in Dentistry; IACSD = Intercollegiate Advisory Committee for Sedation in Dentistry; ASA = American Society of Anesthesiologists.
